# Supplementary material for: Complexome profiling of the Chlamydomonas psb28 mutant reveals TEF5 as an early PSII assembly factor
Source: Plant Cell. 2025 Mar 17;37(6):koaf055. doi: 10.1093/plcell/koaf055 (PMC12164590; doi:10.1093/plcell/koaf055)
Supplement: koaf055_Supplementary_Data [file koaf055_supplementary_data.zip › psb28_interactive.zip!/plots/intensity/qualityPlots/peptides/_Cre10.g450500.t1.1.html]

### Cre10.g450500.t1.1

Trivial name:   
SeqCoverage [median]: 4.60  
Mass: 72.82 kDa  
  
 

| Sequence | MissedCleavage | Mass [Da] | wtIntensity | muIntensity | Score |
| --- | --- | --- | --- | --- | --- |
| GADTAAAVAEESLK | 0 | 1331.66 | 0.00 | 414.45 | 67.39 |
| TLLQSSLAGAQVR | 0 | 1342.76 | 446.92 | 5373.34 | 90.11 |
| VNFGETLR | 0 | 934.49 | 0.00 | 1836.00 | 63.56 |
|  |  |  | 446.9 | 7623.8 |  |
